# Dietary Intake of Gaelic Football Players during Game Preparation and Recovery

**DOI:** 10.3390/sports8050062

**Published:** 2020-05-15

**Authors:** Ciarán Ó Catháin, James Fleming, Michèle Renard, David T. Kelly

**Affiliations:** 1 Department of Sport and Health Sciences, Athlone Institute of Technology, N37 HD68 Athlone, Ireland; m.renard@research.ait.ie (M.R.); davidkelly@ait.ie (D.T.K.); 2 School of Sport Health and Applied Sciences, St Mary’s University, Twickenham TW1 4SX, UK; James.Fleming@stmarys.ac.uk

**Keywords:** Gaelic football, dietary intake, energy expenditure, macronutrients, match-play, game day, recovery

## Abstract

It is well established that dietary intake can influence performance and modulate recovery in field-based invasion team sports such as soccer and rugby. However, very limited research currently exists examining dietary intake of Gaelic football players. This research aimed to examine the dietary intake of Gaelic football players 2 days prior to competition, on game day, and for 2 days post-competition. A five-day paper-based food diary was completed by 45 players (25 elite and 20 sub-elite). Preliminary inspection of diaries eliminated 11 participants, and analysis of Goldberg cut-offs identified 1 player as an under-reporter, leaving 33 players in the final analysis. Playing level had no effect on energy, carbohydrate, or fat intake. Average intake of energy was 2938 ± 618 kcal.day^−1^, carbohydrate was 3.7 ± 1.42 g.kgbm^−1^.day^−1^, and fat was 1.34 ± 0.61 g.kgbm^−1^.day^−1^. However, elite players consumed 24.1% more protein than sub-elite players (2.2 ± 0.67 vs. 1.8 ± 0.62 g.kgbm^−1^.day^−1^). Regardless of playing level, players consumed inadequate amounts of carbohydrate to support optimal performance and recovery and consumed protein and fat in line with general sport nutrition guidelines. Given the unique demands placed on Gaelic football players, it may be necessary to develop nutrition guidelines specific to Gaelic football. Additionally, the design and implementation of Gaelic football-specific education-based interventions may be necessary to address the highlighted nutritional inadequacies.

## 1. Introduction

Gaelic football is an intermittent invasion-based team sport indigenous to Ireland [1,2]. Although categorized as an amateur sport, the high level of competition, physiological characteristics of elite players, and the high volume periodized training, are all reflective of that observed in professional sports [1,3,4]. The popularity of Gaelic football is demonstrated by attendance of up to 80,000 spectators at inter-county games, which are televised live, both nationally and internationally [3]. Games are played on a pitch between 130–145 m in length and 80–90 m in width; approximately 20–40% larger than a soccer pitch [3,5]. Two teams of 15 players compete against each other over two 35-min halves at inter-county level (elite) and two thirty minute halves at club level (sub-elite). Similar to other team sports, the goal is to outscore the opposing team [5]. Points are accumulated by scoring a goal (3 points), or by putting the ball over the crossbar between two uprights (1 point) [3].

Gaelic football match-play is characterized by high-intensity bursts separated by periods of moderate and low intensity activity [6]. On average players cover 116 m.min^−1^, with reports of total distance covered of 8160 ± 1482 m at inter-county level [7], with shorter total distances covered at senior club level due mainly to the shorter total game time. To provide context, these distances are larger than those displayed by elite rugby players (86 m.min^−1^) [8], and similar to those displayed in elite soccer players (118 m.min^−1^) [9]. Of this total distance, 66% is covered by walking or jogging, 12% by striding, and 4% by sprinting [10], with the average high-intensity burst lasting for 4–7 s [11]. This intermittent nature of play results in a caloric expenditure of 58–70 kJ.kg^−1^ body mass per match [12]; with the large variation due to positional differences (midfielders at the high end of the range, full forwards and full backs at the low end, and half backs and half forwards in between) [12]. Therefore, with an average body mass of 84 kg (±7) observed in elite Gaelic football [5], the typical caloric expenditure per match is approximately 1164–1405 kcal. 

To maximize performance, it is necessary that peri-match nutrition intake is reflective of the energy cost of match-play [13]. Currently, research examining nutrition strategies in Gaelic football are severely limited [1,3,14]. However, it has been identified that during periods of training, player’s dietary intake does not meet the energy requirements recommended for team sport athletes [15]. During pre-season, inter-county players consume on average 3.6 ± 0.7 g.kg^−1^ per day of carbohydrate [14], which is below the lower end of the recommendation of 5–10 g.kg^−1^ per day suggested for optimal recovery and performance [13,14,16]. Furthermore, research demonstrates that this low carbohydrate intake remains stable throughout the week, despite large variations in training load intensity (Pitch vs. Gym vs. Rest) [14], and is similar to patterns observed in professional rugby league players [17]. Consequences of a chronically low carbohydrate intake range from a reduced capacity to use carbohydrate as a fuel, to increased muscle breakdown and reduced immune function [18], all which have negative performance and health related implications. This low carbohydrate intake appears to coincide with protein and fat intake in excess of recommendations, with players averaging 2.1 ± 0.5 g.kg^−1^ of protein per day, and 37.5 ± 3.6% of total daily energy intake of fat per day, during pre-season [14]. Although only marginally above recommendations (1.2–2 g.kg^−1^ per day protein, and 20%–35% total energy intake fat) [19], higher protein and fat intake has been shown to negatively influence total carbohydrate intake in professional soccer players [20].

A similar pattern has been observed in preparation for Gaelic football match-play, where players do not consume sufficient carbohydrate on the day prior to matches (by up to 61%) when compared to guidelines (7–10 g.kg^−1^), and in similar fashion to pre-season, players consume excessive fat and protein, possibly having a limiting effect on carbohydrate consumption [1]. Therefore, it appears that nutrition strategies during training and prior to competition do not prepare players to compete optimally. To date, there is currently no research examining the dietary practice of Gaelic footballers during the recovery period after competitive match-play or comparing dietary intake between playing levels around competition. Previous research has demonstrated differences in dietary intake between elite and sub-elite Australian football players and soccer players [21]. With this in mind, the development of different education strategies specific to playing level may be required, and warrants investigation in Gaelic football. 

Therefore, the aim of this research was to examine the energy and macronutrient intake of Gaelic footballers for 2 days prior to competition, on game day, and for 2 days post-competition. This will be used to identify if players meet current dietary recommendations to optimize performance and recovery, and to examine if dietary intake changes during these 5 days. Secondly, this study aimed to examine if playing level influences dietary intake by comparing dietary intake of inter-county (elite) players against senior club (sub-elite) players. 

## 2. Materials and Methods 

### 2.1. Participants and Study Design

Forty-five male Gaelic football players (mean age: 24.8 ± 4.6 years; mean height: 1.81 ± 0.05 m; mean body mass: 82.3 ± 7.1 kg) between the ages of 18–30 years were recruited. This included 25 inter-county players (elite) and 20 senior club players (sub-elite). All participants were older than 18 years of age, on a senior club panel or inter-county panel, and at the time of data collection were injury, illness, and suspension free, and thus available for selection for competitive match-play [1]. All data collection was completed in accordance with the declaration of Helsinki and the ethics review board at St. Mary’s University, London, granted ethical approval.

All participants were required to complete a 5-day food diary. This included recording of food intake for 5 consecutive days; 2 days prior to game day, game day, and two days’ post-game day. For all participants the first and fifth days were training days, with the second day and fourth day acting as rest days to prepare and recover for/from game day, respectively (Table 1). All data was collected around early season competitive league games. Height and weight of each participant was recorded on the first and last day of data collection, with average values reported.

### 2.2. Anthropometrics

Weight was measured using an electronic digital scale (Seca, Hamburg, Germany) and recorded to the nearest 0.1 kg. Participants were weighed without shoes and in undergarments. Height was recorded to the nearest centimeter (0.01 m) and measured using a stadiometer (Seca, Hamburg, Germany), without shoes and with the head positioned in the Frankfurt plane position [22].

### 2.3. Dietary Records

As used in previous studies, dietary intake was assessed using paper-based food diaries [1,14]. For this purpose, all players completed a 5-day EPIC (European Prospective Investigation of Cancer) food diary [23]. When compared to a 16-day weighed record, the EPIC food diary displayed no significant difference for reporting macronutrients [23]. Food Diary collection periods typically range from 3–7 days, and present a paradigm where a greater number of days’ increases accuracy, however decreases compliance and increases risk of altering habitual eating behaviors [24]; thus 5 days was selected in an attempt to balance these factors. Each EPIC food diary contains four pages of space where participants record all food and drink consumed across 7 meal options (before breakfast, breakfast, between breakfast and lunch, lunch, between lunch and dinner, dinner, and after dinner) [23,25]. Players were instructed to weigh and record all food and drink prior to consumption and after consumption in order to determine actual food/drink consumed [26], where possible. In scenarios where a scale was not available players were instructed to use household measures (such as tablespoon or cups), to refer to the 17 pictures at the start of the EPIC diary that depict commonly used foods along with portion sizes [23,25], or to record weights from packaging [27]. Participants were requested to note the time of consumption, the type of food/drink, the brand, and the ingredients included in homemade recipes, and the method of cooking [1]. Participants were advised to record intake as close to the time point of consumption as possible to reduce recall error. In conjunction with space for meals, each day of data entry in the EPIC diary includes a checklist of commonly forgotten foods and the last pages of the diary includes a questionnaire that assesses types of milk, bread, oils, spreads, as well as brands, in order to aid data entry in cases where players did not include enough detail in their diary [27]. 

In an attempt to increase adherence, regular messages of encouragement were sent to both individuals and via WhatsApp groups throughout the data collection period [28]. After data collection, each player completed an interview with the primary researcher in order to review unclear descriptions or omissions [14]. Participants who submitted an incomplete or unsatisfactory EPIC food diary were not included in the analysis. All data from the food diaries was assessed using Nutritics dietary analyses software (v5.099, Nutritics, Dublin, Ireland). Dependent variables are reported relative to body mass and include energy, macronutrient, and alcohol intake. Data was extracted for each day of data collection for energy and macronutrients, and game day only for alcohol. Furthermore, game day intake was separated into pre-game intake and post-game intake to assess game preparation and recovery strategies. Pre-game intake included all dietary intake before kick-off on game day, and post-game intake included everything after the final whistle on game day. 

The ratio of energy intake to basal metabolic rate (EI:BMR) was calculated at an individual level in order to assess the validity of collected dietary intake data [29,30]. To do this, mean energy intake was calculated from the 5-day food diaries of each participant and the Harris–Benedict equation was used to estimate BMR [31]. This is the recommended equation for calculation of BMR in athletic populations when lean body mass is unknown [32], and has been used in similar research for the same purpose [1]. A physical activity level of 1.66 was used to determine the level of agreement, based on assessment of activity across the 5-day period. Finally, lower and upper confidence intervals (95%) were calculated using the equations provided by Black [29]. Participants with EI: BMR ratios below or above these 95% confidence intervals were deemed as under- and over-reporters respectively, and were excluded from the analysis.

### 2.4. Statistical Analysis

All parametric data is reported as means with standard deviations. Statistical analysis was completed using IBM SPSS statistical software (v23.0 for windows, IBM corporation, Armonk, NY, USA). Normality of data was assessed using the Shapiro–Wilks test, with *p* > 0.05 used as the threshold for determination of normal distribution. Independent sample *t*-tests were completed to compare height, weight, age, and BMR between playing levels (inter-county vs. senior club). Multiple 2 × 5 (playing level × day) between–within repeated measure ANOVAs were used to examine the change in dependent variables (energy, carbohydrate, protein, and fat intake) across the 5 days (within effect: training 1, pre-game day, game day, post-game day, and training 2) of recorded dietary intake and to examine the effect of playing level (between effect: inter-county vs. senior club). Similarly, multiple 2 × 2 (playing level × game-day macronutrient timing) between–within repeated measure ANOVAS were used to compare energy and macronutrient intake before the game and after the game (within effect) and to compare differences between playing levels (between effect) on game day data. Assumptions of sphericity and homogeneity of variance were assessed using Mauchly’s and Levene’s test, respectively. For each, an alpha level of *p* < 0.05 determined a violation of the assumption. Finally, a Mann–Whitney U test was used to compare alcohol intake between playing levels on game day.

## 3. Results

### 3.1. Participant Characteristics and Mean Dietary Intake

Participant characteristics and comparison of mean macronutrient intake relative to sports nutrition recommendations can be observed in Table 2 and Table 3, respectively. Preliminary inspection eliminated 11 participants due to incomplete food diaries. Subsequent analysis of Goldberg cut-offs identified 1 player as an under-reporter, 33 acceptable reporters, and no over-reporters, thus leaving a sample of 33 players for the remaining dietary intake analysis. A Shapiro–Wilks test determined that all data was normally distributed (*p* > 0.05), apart from alcohol intake on game day (*p* > 0.05). Furthermore, assumptions of homogeneity of variance and sphericity were met for all ANOVA’s completed (*p* > 0.05). Independent sample *t*-tests indicated no significant difference between playing levels for height (*p* = 0.59), weight (*p* = 0.64), age (*p* = 0.27), and BMR (*p* = 0.45). A Mann–Whitney U test identified no significant difference for alcohol intake between playing levels on game day (U = 181, z = 1.91, *p* = 0.084). Mean Intake for the entire sample was 0.46 ± 0.83 g.kg^−1^, with 18 out of the 33 players not consuming alcohol. Of the 15 that did consume alcohol, mean intake was 1.16 ± 0.98 g.kg^−1^.

### 3.2. Dietary Intake: Comparison of Days and Playing Level

**Figure 1:**
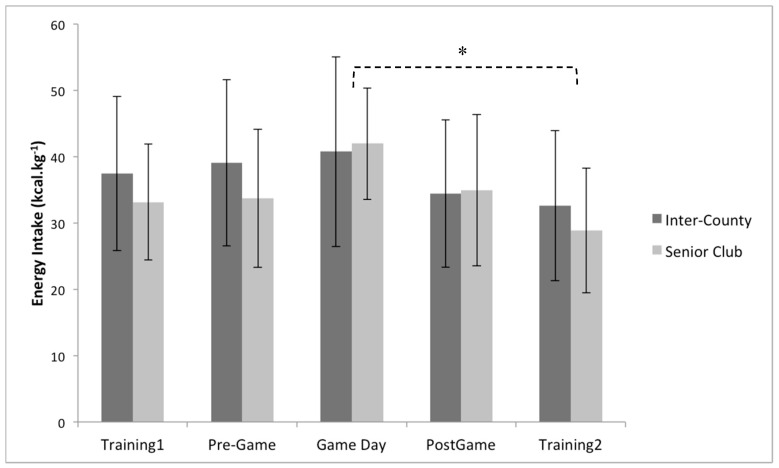
Between group (playing level: inter-county (n = 19) vs. senior club (n = 14)) and within group (comparison of days) analysis for total energy intake. * Indicates a significant main effect of time (*p* < 0.05) between Game day and Training2. Values are displayed as means with standard deviations.

**Figure 2:**
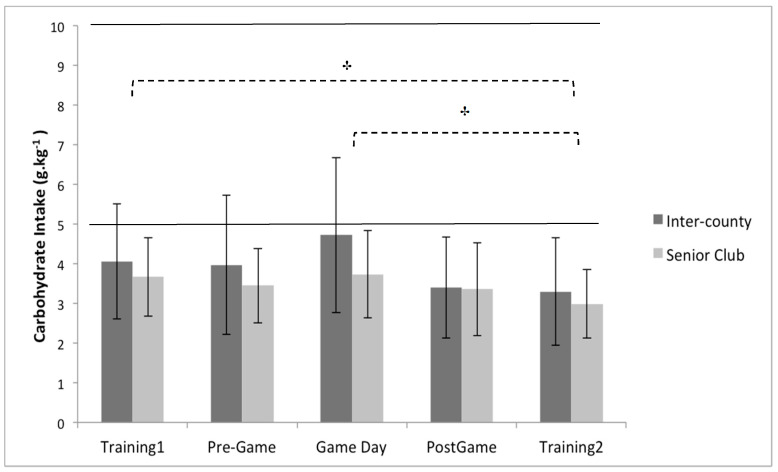
Between group (playing level: inter-county (n = 19) vs. senior club (n = 14)) and within group (comparison of days) analysis for relative carbohydrate intake. † Indicates a significant main effect of time (*p* < 0.05) between Training 1 and Training 2, and Game Day and Training 2. Values are displayed as means with standard deviations. Area between the solid lines represent the sports nutrition guidelines.

**Figure 3:**
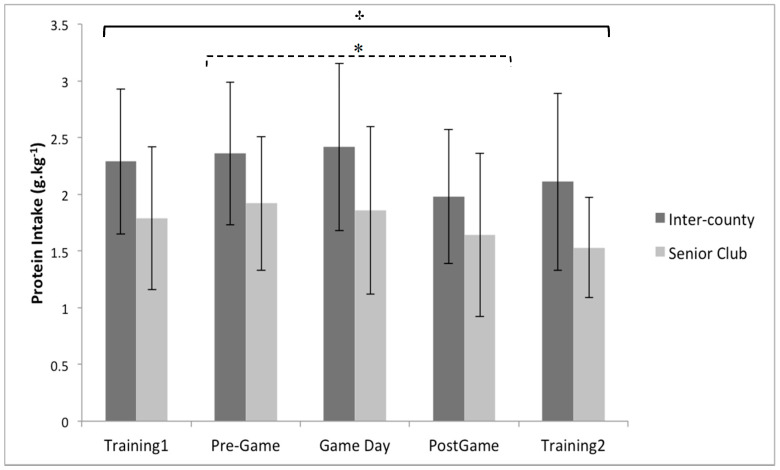
Between group (playing level: inter-county (n = 19) vs. senior club (n = 14)) and within group (comparison of days) analysis for relative protein intake. * Indicates a significant main effect of time between pre-game and post-game. † indicates a significant main effect of playing level. Values are displayed as means with standard deviations.

Multiple 2 × 5 (playing level × day) between–within repeated measures ANOVA’s identified no significant interaction effect between playing level (Inter-County vs. Senior club) and day (training 1, pre-game, game day, post-game, and training 2) for daily, energy intake (*p* = 0.47, partial eta squared = 0.03), carbohydrate intake (*p* = 0.53, partial eta squared = 0.03), protein intake (*p* = 0.26, partial eta squared = 0.01), and fat intake ( *p* = 0.63, partial eta squared = 0.02) (Figure 1, Figure 2 and Figure 3). Similarly, no significant main effects were observed for playing level for daily, energy intake (*p* = 0.40, partial eta squared = 0.02), carbohydrate intake (*p* = 0.18, partial eta squared = 0.06), and fat intake (*p* = 0.82, partial eta squared = 0.002). However, a significant main effect for playing level was identified for daily protein intake (*p* < 0.05, partial eta squared = 0.21), with inter-county players consuming on average 24.1% per day more than senior club players (Figure 3).

Daily energy intake demonstrated a significant main effect for day (training 1, pre-game, game day, post-game, and training 2), (*p* < 0.001, partial eta squared = 0.17), with post hoc pairwise comparisons indicating a significant difference between game day and training 2 (*p* < 0.05, game day > training 2 by 29.5%) (Figure 1).

There was a significant main effect for day when assessing daily carbohydrate intake (*p* < 0.05, partial eta squared = 0.13), with post-hoc pairwise comparisons indicating that players consumed more carbohydrate on training 1 than training 2 by 20.6% (*p* < 0.05), and on game day than training 2 by 29.4% (*p* < 0.05) (Figure 2). In conjunction with the playing level differences described above, the ANOVA analysis also demonstrated a significant main effect for day when assessing daily protein intake ( *p* < 0.05, partial eta squared = 0.10), with pairwise comparisons indicating that protein intake was greater on pre-game day than post-game day by 16.7% (*p* < 0.05) (Figure 3). In comparison to the other macronutrients, the ANOVA analysis demonstrated no significant main effect for day when assessing daily fat intake (*p* = 0.13, partial eta squared = 0.06).

### 3.3. Comarison of Pre-Game Intake and Post-Game Intake on Game Day

**Figure 4:**
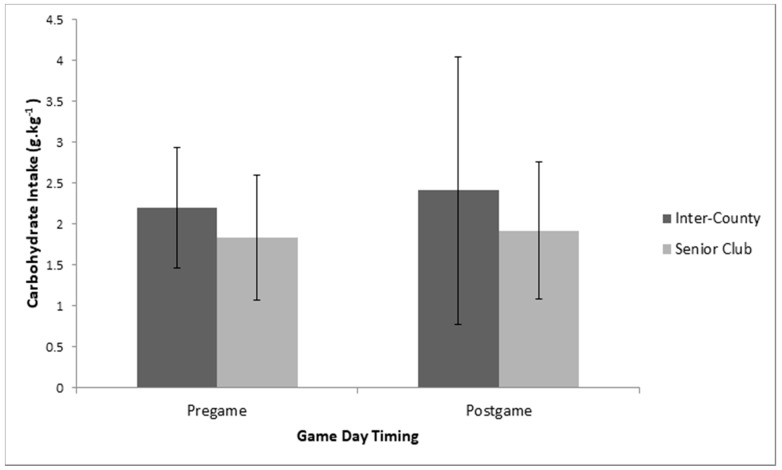
Between group (playing level: inter-county (n = 19) vs. senior club (n = 14)) and within group (Pre-game vs. Post-game) analysis for relative carbohydrate intake on Game Day.

**Figure 5:**
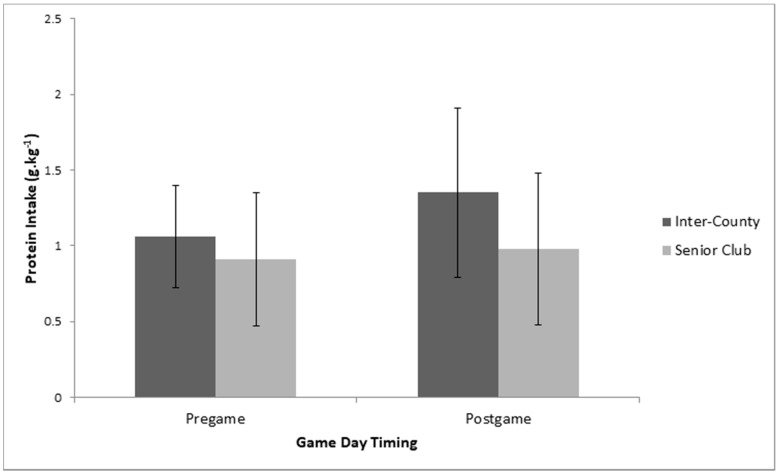
Between group (playing level: inter-county (n = 19) vs. senior club (n = 14)) and within group (Pre-game vs. Post-game) analysis for relative protein intake on Game Day.

Multiple 2 × 2 (Playing Level *Macronutrient game-day timing) between–within repeated measures ANOVA’s indicated no significant interaction effects between playing level and timing of game-day macronutrient intake (pre-game vs. post-game) for carbohydrate (*p* = 0.81, partial eta squared = 0.002), protein (*p* = 0.26, partial eta squared = 0.04), and fat intake (*p* = 0.13, partial eta squared = 0.07). Similarly, there were no significant main effects for playing level or macronutrient timing on game day for carbohydrate (Playing level: *p* = 0.13, partial eta squared = 0.01; Timing:, *p* = 0.16, partial eta squared = 0.06) (Figure 4, protein (Playing level: *p* = 0.06, partial eta squared = 0.11; Timing: *p* = 0.08, partial eta squared = 0.09) (Figure 5, or fat intake (Playing level: *p* = 0.36, partial eta squared = 0.01; Timing: *p* = 0.10, partial eta squared = 0.03).

## 4. Discussion

The aim of this study was to examine the dietary intake of Gaelic football players for the 2 days prior to a game, game day, and 2 days post-game in order to assess whether player’s intake was in line with current sports nutrition recommendations and if intake varied from day to day. Secondly, this study aimed to assess if intake differed between playing level (inter-county vs. senior club). To the authors knowledge, this is the first study to examine post-game nutrition strategies in Gaelic footballers and to compare the nutrition intake of inter-county and senior club players. 

Findings indicate that Gaelic football players do not meet sports nutrition guidelines. Although there was some day-to-day variation in dietary intake, the pattern of variation does not appear to be in a manner that reflects purposeful nutritional periodization to optimize game preparation and recovery. Although inter-county players (elite) consumed more protein than senior club players (sub-elite) by 24%, there was no difference between playing levels for carbohydrate, fat or total energy intake. Mean total energy intake for the 5 days of recorded data was similar to that reported for Gaelic footballers in previous research, as were carbohydrate, fat, and protein intake [1,14,15]. 

Although players consumed on average 29.5% more calories on game day than on training 2, there was no significant difference between all other days. Examination of data appears to indicate this was largely due to a reduction in intake on training day 2 as opposed to a purposeful effort to consume more on game day. It is well established that players should adjust their energy and macronutrient intake in a periodized fashion to support training and competition demands in a manner that optimizes performance and health [13,14,19]; however this does not appear to be the case. Previous research that reported similar, but slightly higher, energy intakes during pre-season in Gaelic football players (2938 ± 618 kcal vs. 3282 ± 483 kcal), also measured energy expenditure using wearable sensors and identified that players were consistently in an average energy deficit of 460 ± 503 kcal during a period that included rest days, gym sessions, and pitch sessions [14]. Although energy expenditure was not measured in the current study, consideration of the data presented by O’Brien et al [14], in conjunction with previous reports detailing high energy demands of competitive match-play (1164–1405 kcal per game) [12], it is possible that players in the current study did not consume sufficient calories to optimize performance and recovery, potentially due to low relative carbohydrate intake. 

With regard to carbohydrate intake, there was no significant difference between playing levels; however players consumed more carbohydrate on training day 1 and game day, than training day 2 by 20.6% and 29.4%, respectively. Players consumed the highest amount of carbohydrate on game day with an average intake of 4.3 ± 1.7 g.kg^−1^, and an average across the 5 days of 3.7 ± 1.7 g.kg^−1^. These values are similar to those previously reported during pre-season [14] and before competitive match-play [1] in Gaelic football, as well as other team sports such as soccer and rugby [33,34], and fall short of the sports nutrition guidelines of 5–10 g.kg^−1^ for training days [14,19], and 7–10 g.kg^−1^ on the day prior to competition [13,19]. Intermittent high-intensity team sports, such as Gaelic football, are highly reliant on carbohydrate as a fuel source to optimize and sustain maximal performance [3,35,36]; thus the current study indicates that players are not optimally prepared for competitive match-play. To provide context, previous research indicates that players who consume carbohydrate in line with pre-competition guidelines perform 33% more high-intensity bursts in soccer [37], and cover larger total distances by 30% in ice hockey [38]. Furthermore, ice hockey players exhibit a reduction in total distance skated in the final period (relative to the first) by 14% when carbohydrate intake is low (relative to guidelines), whereas those who consume carbohydrate in line with guidelines demonstrate an increase in distance covered during this period (by 11%) [38]. Similar reductions in total distance covered, and high speed running distance have been previously demonstrated in the latter quarters of game play in Gaelic football [7]. It is, therefore, possible that inadequate carbohydrate intake leading into game day, as demonstrated in the current study and previous research [1], may be a contributing factor to these observed performance decrements.

On game day, guidelines dictate that players should consume 1–4 g.kg^−1^ of carbohydrate in the 1–4 h prior to match-play [13,19]. In agreement with previous literature [1], players had no problem meeting these guidelines with an average intake of 2 g.kg^−1^, and as previously suggested may point to a misguided perception among teams that increased carbohydrate intake on game day alone is sufficient to maximize performance [1]. Despite this, total carbohydrate intake on game day (4.3 ± 1.7 g.kg^−1^) was below recommendations and may be explained by an intake of 2.2 g.kg^−1^ in the period between the end of the game and the end of the day. Optimal post-match nutrition intake is largely dictated by the time frame between two subsequent performances, or between a match and subsequent training sessions, where the activity is carbohydrate dependent [13,16,19]. During the 2 h post-exercise muscle appears to demonstrate an increased ability to store glycogen (by up to 57%) [16,39]. Typical rates of glycogen resynthesize thereafter are approximately 5% per hour [19,22]. Therefore, early intake of carbohydrate in the recovery period, of 1–1.2 g.kg^−1^ per hour for the first 4–6 h, will maximize the speed of refueling [19,39]. However, if recovery is not time-sensitive, and longer time periods exist between successive matches or a match and a subsequent training session (>24 h), once total carbohydrate intake is sufficient (5–10 g.kg^−1^ per day), it appears athletes can self-select the timing of intake, without impairing glycogen repletion [16]. To this end, recovery was not time-sensitive in the current sample; however, players failed to meet the recommendations for total daily carbohydrate intake.

In summary, the carbohydrate intake in Gaelic football players observed in preparation and recovery from training and competition in the current study and previous research appears inadequate [1,14]. Long-term, this may lead to chronic low carbohydrate availability, resulting in reduced exercise intensity [40,41], reduced training volume between games and during pre-season [42], increased muscle breakdown [18], decreased immune function [43], and a reduced ability to use carbohydrate as a fuel source [44]. 

Inter-county (elite) players consumed significantly more protein than senior club players (sub-elite) by an average of 24.1%, which is in line with previous research demonstrating a similar pattern between playing levels in Australian football [21]. All players appeared to consume more protein on the day before game day (pre-game) than the day after game day (post-game) by 16.7%, with no difference between any of the other days. Average daily protein intake for inter-county players was 2.2 g.kg^−1^ per day, which is similar to values previously reported during pre-season training (2.1 g.kg^−1^ per day) [32] and prior to competition (2.0 g.kg^−1^ per day) [1], whereas senior club players consumed an average of 1.8 g.kg^−1^ per day. This indicates that senior club players fall within the recommended sports nutrition guidelines of 1.4–2 g.kg^−1^ of protein per day [19,45], with inter-county players consuming slightly more than recommended. Protein intake is essential for recovery, and to support maintenance or increases in muscle mass [19,46], by reducing muscle protein breakdown and promoting muscle protein synthesis [19,46]. In addition, co-ingestion of protein with carbohydrate post-exercise may enhance glycogen repletion [19], due to the positive effect that protein intake has on reducing muscle damage [47,48], as muscle damage may negatively influence glycogen resynthesis [16]. However, over consumption of protein and fat has previously been associated with reduced carbohydrate intake in elite soccer players [20], and may partly account for the inadequate carbohydrate intake observed in inter-county players across all days, and for senior club players on the day before game day. It is suggested that players should consume less protein one day before competition in order to facilitate the higher carbohydrate intake (7–10 g.kg^−1^) required to optimize glycogen storage [19]; however, average intake on this day was 2.3 g.kg^−1^ for inter-county players, and 1.9 g.kg^−1^ for senior club players. It has been suggested that higher protein intakes of up to 2.3 g.kg^−1^ per day may be required in scenarios where players are attempting to preserve muscle mass while in an energy deficit [49] or during periods of intense and/or high volume training [50]. Therefore, these higher protein intakes may be appropriate during pre-season where body re-composition is a major goal [14] and training load is higher [50]. However, players should adopt a periodized approach where priority shifts from maximizing training adaptation and body composition during pre-season, to optimizing performance during periods of competition [19]. From a nutritional perspective, this may mean reducing protein to favor higher carbohydrate intake during periods of competition. Average fat intake in the current sample was 33.4 ± 4.2% of daily energy intake, which is similar to previous investigations [1,14] and on the higher end of the scale for sports nutrition recommendations (20–35% of total daily energy intake) [19]. There was no significant difference between playing levels or between days for fat intake in the current sample. Although within the recommendations, higher levels of fat intake are associated with reduced carbohydrate consumption [20]. In line with previous recommendations, reducing fat intake may allow players to focus on increased carbohydrate intake in order to optimize performance and recovery [1,14,19]. Furthermore, fat intake in close proximity to competition is associated with gastrointestinal discomfort and delayed gastric emptying [51,52], and should be reduced on game day to avoid such issues. 

There was no significant difference between playing levels for alcohol intake on game day and intake ranged from 0.0–3.5 g.kg^−1^, which is in line with previous literature that observed binge drinking of alcohol post-game in Australian football teams [53]. Although not all players consumed alcohol post-game (15/33 players did), acute alcohol intake has been shown to displace carbohydrate intake during and on the day after consumption [54], and may partly explain the reduced intake observed in the current study. Alcohol intake of 1.5 g.kg^−1^ has been shown to impair carbohydrate and lipid metabolism when consumed after prolonged exercise, with evidence suggesting an impairment in glycogen resynthesis [54]. This may be compensated for with adequate carbohydrate intake [54]; however, this is not the case in the current sample. Furthermore, consumption of 1 g.kg^−1^ of alcohol has been shown to increase losses in strength associated with muscle damage following eccentric exercise by up to 22% [55]. Finally, habitual alcohol drinkers display an increased injury risk relative to non-drinkers (55% vs. 24%) [56]. Thus, players should be educated on the effects of alcohol and encouraged to reduce intake to optimize performance and recovery.

Although the current study offers an interesting insight into the dietary habits of inter-county and senior club level Gaelic football players there are several limitations that should be considered. First and foremost, the measurement of dietary intake must be assessed with vigilance due to the inherent limitations of underreporting and misreporting associated with self-report data [57,58]. This is a particular issue in athletic populations where the increased energy demands associated with training may increase the number of meals or eating occasions, resulting in more opportunities to misreport [59]. Subsequently a bias of up to 36% has been reported for energy intake in athletes [60,61,62]. Although the ratio of energy intake to BMR was assessed in order to identify under- or over-reporters [29], both BMR and Physical Activity Level (PAL) were estimated. To increase the accuracy of this validation process, future work should include objective measures of energy expenditure, such as the use of SenseWear armbands [14]. Furthermore, the current study employed a relatively long data collection period of 5 days, which may have impaired compliance [40]. Despite this, it is encouraging that the current study reports similar energy intake values as previous research assessing dietary intake in Gaelic football [1,14]. In addition, the current study is limited by sample size, and by the fact that it only represents one time period during the year. Dietary intake was recorded early in the competition calendar and it is possible that it is not representative of nutrition practices at the more competitive end of the season. Furthermore, most inter-county players came from the same team, as did senior club players. Therefore, inclusion of a larger sample size from multiple teams may provide a more accurate portrayal of the population. The sports nutrition guidelines presented as a comparison point throughout the above discussion, are general guidelines that are not specific to Gaelic football. Therefore, the development of specific guidelines for Gaelic football nutritional intake during competition season, as reported for pre-season by O’Brien et al [14], is required. Furthermore, future work should consider food sources and dietary quality. This may be particularly important with regard to protein, where leucine content plays an important role in stimulating MPS [45].

In conclusion, inter-county and senior club Gaelic football players appear to consume carbohydrate below that recommended to support optimal performance and recovery from competitive game play and training. This may be partly explained by consumption of protein in excess of guidelines (inter-county), or at the higher end of guidelines (senior club), thus negatively influencing carbohydrate consumption. Although day-to-day variation in intake was observed for protein and carbohydrates, this does not appear to be in a manner that reflects planned nutritional periodization. Inter-county players consumed significantly more protein than senior club players, potentially pointing to an over-emphasis on protein intake in inter-county Gaelic football environments. Thus, the current study highlights a potential need for nutrition-based education and interventions in both inter-county and senior club Gaelic footballers. 

## Figures and Tables

**Table 1:** Description of data collection days.

Food Diary Day	Data Label	Description
Day 1	Training 1	Light Intensity Pitch Session
Day 2	Pre-game	Rest
Day 3	Game day	Mid-Afternoon Game
Day 4	Post-game	Rest
Day 5	Training 2	Medium-Intensity Pitch Session

Note: Data labels are used to reference days 1–5 throughout document.

**Table 2:** Participant Characteristics.

Characteristics	Inter-County (n = 19)	Senior Club (n = 14)	Total (n = 33)
Height (m)	1.80 ± 0.05	1.81 ± 0.05	1.81 ± 0.05
Mass (kg)	82.9 ± 6.5	81.5 ± 8	82.3 ± 7.1
Age (Years)	24 ± 3.8	25.9 ± 5.9	24.8 ± 4.9
BMI	25.5 ± 1.6	24.8 ± 2.6	25.2 ± 2.1
BMR (kcal)	1947 ± 102	1919 ± 103	1935 ± 102
Mean EI	3038 ± 726	2802 ± 421	2938 ± 618
EI: BMR	1.6 ± 0.4	1.5 ± 0.2	1.5 ± 0.3

Note: All Values are reported as means ± standard deviations. BMI = Body Mass Index; BMR = Basal Metabolic Rate; EI= Energy Intake.

**Table 3:** Mean Daily Macronutrient Intake in comparison to Sports Nutrition Recommendations.

Macronutrient		Dietary Intake	Sports Nutrition Recommendations
Carbohydrate	g.kg^−1^ Body Mass	3.7 ± 1.4	5–10 g.kg^−1^ per day
	% Total Energy Intake	41.3 ± 1.7	
Protein	g.kg^−1^ Body Mass	2.0 ± 0.7	1.2–2 g.kg^−1^ per day
	% Total Energy Intake	22.6 ± 1.4	
Fat	g.kg^−1^ Body Mass	1.3 ± 0.6	20–35% Total Energy Intake
	% Total Energy Intake	33.4 ± 4.2	

Note: Percentages do not total to 100%, as alcohol intake was not included. Recommendations based on those previously presented [13,19]. All Values are reported for the full sample (inter-county + senior club) and are reported as means ± standard deviations.
